# SRplot: A free online platform for data visualization and graphing

**DOI:** 10.1371/journal.pone.0294236

**Published:** 2023-11-09

**Authors:** Doudou Tang, Mingjie Chen, Xinhua Huang, Guicheng Zhang, Lin Zeng, Guangsen Zhang, Shangjie Wu, Yewei Wang

**Affiliations:** 1 Department of Respiratory and Critical Care Medicine, the Second Xiangya Hospital, Central South University, Changsha, Hunan, China; 2 Shanghai NewCore Biotechnology, Minhang District, Shanghai, China; 3 Shenzhen Ping’an Financial Technology Consulting Co. Ltd, Pudong New District, Shanghai, China; 4 Department of Hematology, the Second Xiangya Hospital, Central South University, Changsha, Hunan, China; University of Nebraska-Lincoln, UNITED STATES

## Abstract

Graphics are widely used to provide summarization of complex data in scientific publications. Although there are many tools available for drawing graphics, their use is limited by programming skills, costs, and platform specificities. Here, we presented a freely accessible easy-to-use web server named SRplot that integrated more than a hundred of commonly used data visualization and graphing functions together. It can be run easily using all Web browsers and there are no strong requirements on the computing power of users’ machines. With a user-friendly graphical interface, users can simply paste the contents of the input file into the text box according to the defined file format. Modification operations can be easily performed, and graphs can be generated in real-time. The resulting graphs can be easily downloaded in bitmap (PNG or TIFF) or vector (PDF or SVG) format in publication quality. The website is updated promptly and continuously. Functions in SRplot have been improved, optimized and updated depend on feedback and suggestions from users. The graphs prepared with SRplot have been featured in more than five hundred peer-reviewed publications. The SRplot web server is now freely available at http://www.bioinformatics.com.cn/SRplot.

## Introduction

Data visualization and presentation are essential parts of scientific publications. A number of packages and software have been developed for graph production. Many of these tools are written in programming languages (e.g., R, Python, and Perl) and rely on coding knowledge and command-line environments, which are difficult for wet-lab researchers [1]. In addition, some of the tools are difficult to install, lack easy-to-use interfaces or are not user friendly, especially for wet-lab biologists or those unfamiliar with programming languages [2, 3]. Moreover, most bioinformatics tools or packages are developed for specific tasks, and scientists are always required to use several, or even dozens of different packages or tools sequentially to prepare all graphs used for a single paper [4]. As a result, different software and packages must be purchased and installed, or downloaded. When the software run locally, they always have high computational requirements. In some cases, modification of graph features and annotations and customizing the output are still quite sophisticated for an average user [5]. Some commercial products, including Microsoft Excel, Origin and Graphpad Prism, are available at this time. They always need hundreds of US dollars per license [1]. Recently, there is an increasing number of freely available graphing and visualization tools. However, most of the tools are not designed specifically for biomedical researchers. Based on the above reasons, we decided to create an easy-to-use, online plot tool.

**Fig 1:**
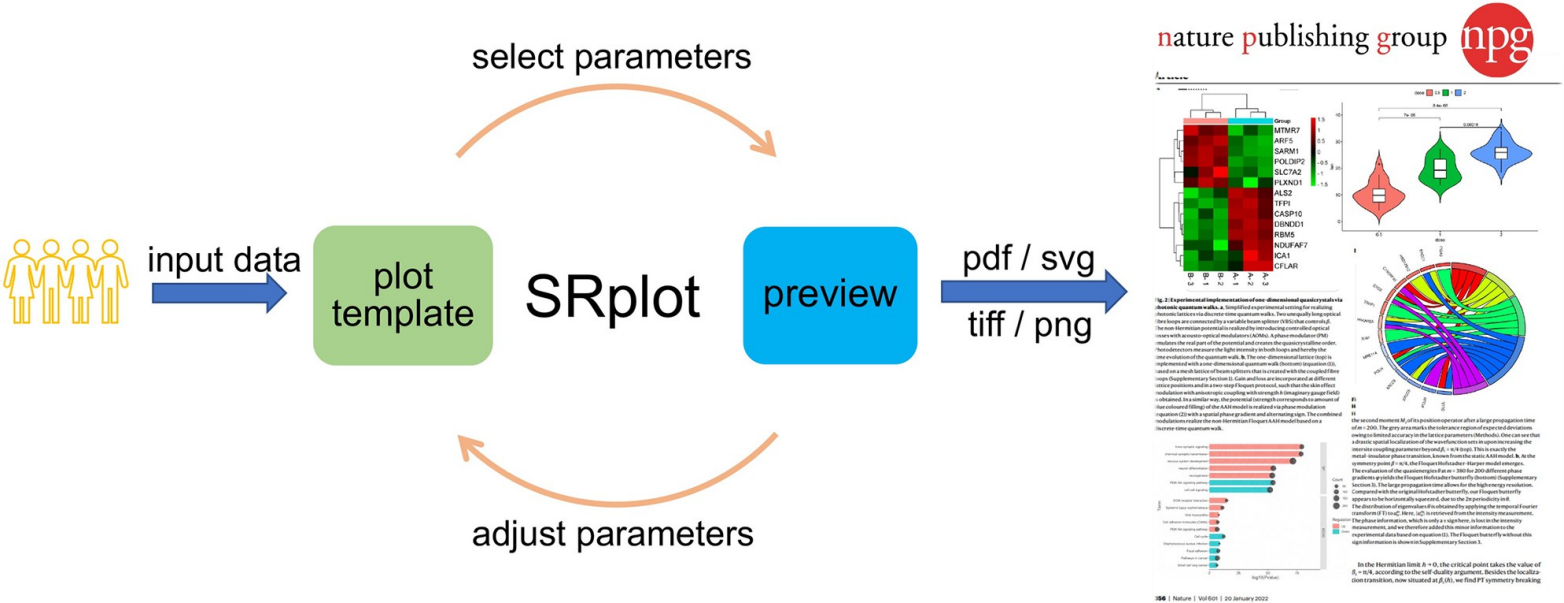
Workflow of SRplot.

Here, we present SRplot (Scientific and Research plot tool) that aims to integrate kinds of the commonly used functions within life science into a comprehensive online plot tool. Through its intuitive graphical user interface and associated example files, SRplot allows users to quickly prepare a wide variety of plots for many different graph types. The following are the features of SRplot: 1) it is an out-of-the-box web tool: users without coding skills could use to create many kinds of graphs; 2) example data and formats are offered to help the user to specify input parameters; 3) it provides several customizable options for changing features of the graph’s appearance; 4) the high-resolution plots can be downloaded in one of the preferred file formats and directly used in publications (Fig 1). In the past two years, the tool has attracted over 46 000 stable users and has already been cited by more than 550 scientific publications (Google Scholar data). Many of these users actively provide informative feedback and suggestions, which has markedly enhanced the functionality, robustness, and features of SRplot. The SRplot web server is now freely available at http://www.bioinformatics.com.cn/SRplot.

## Methods

Functions in SRplot are coded with Python and/or R. Up to now, more than 120 modules are available in SRplot, covering the plots most commonly used for biomedical and bioinformatical data visualization and graph production. Although most of these functions are not originally developed as part of SRplot, they have been optimized and upgraded. In addition, SRplot contains some commonly used databases on the server, such as genomes/transcriptomes/GO [6] /KEGG [7] databases from human/mouse/rat, and the TCGA data (in MAF format).

## Results

### Main functions

**Fig 2:**
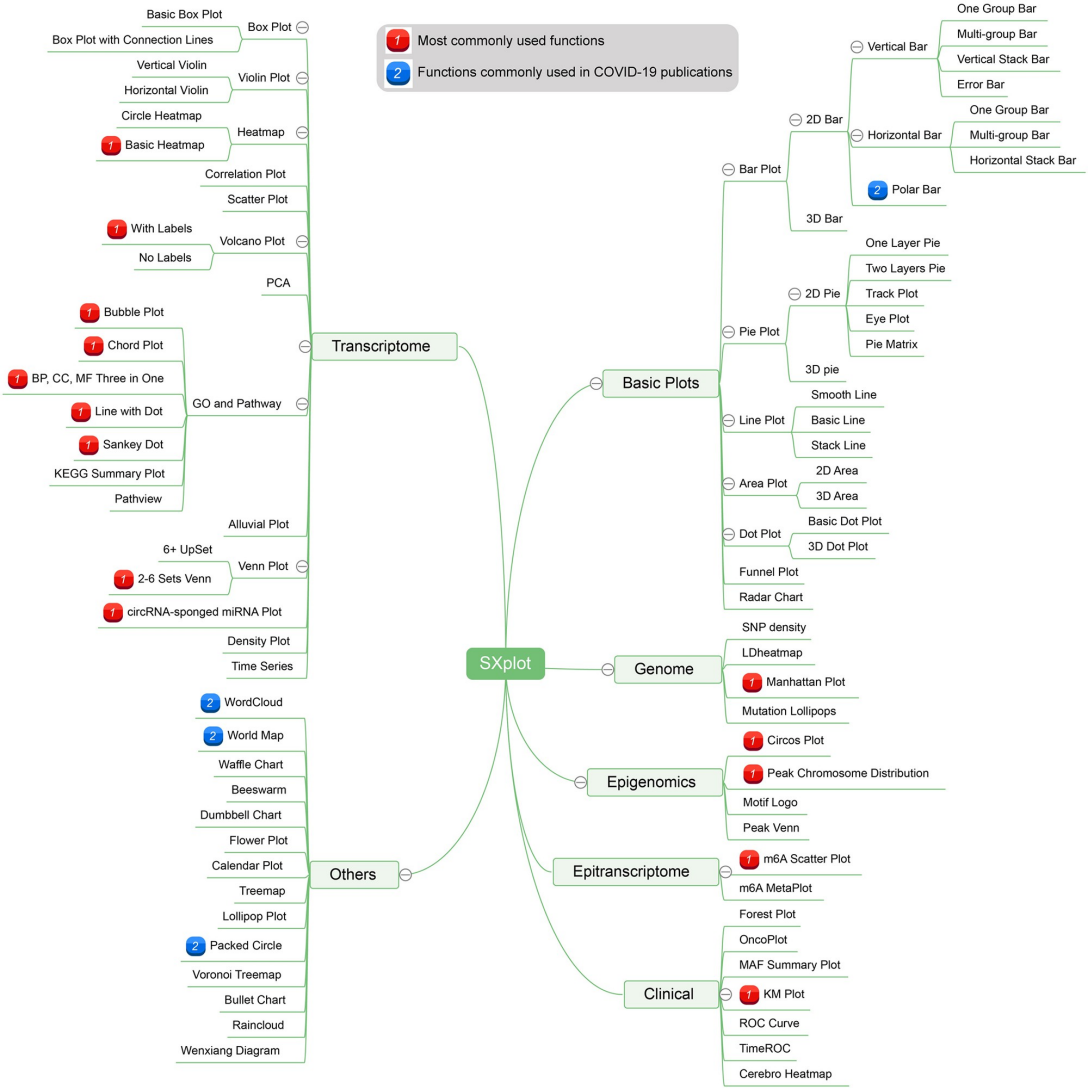
Outline of functions in SRplot.

**Fig 3:**
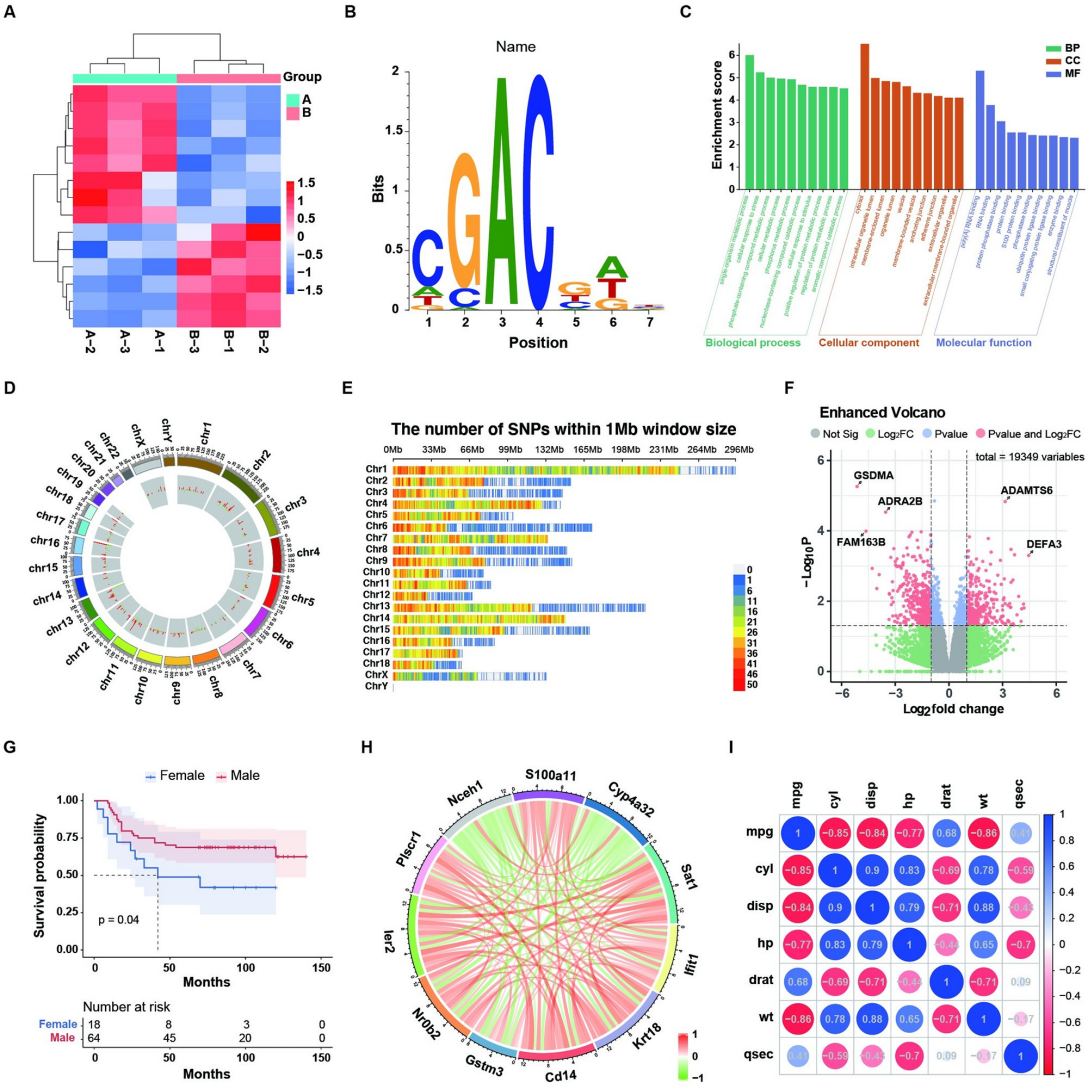
Examples of the most commonly used functions in SRplot. (A) Cluster heatmap; (B) motif logo; (C) Enrichment GO term (BP/CC/MF); (D) Two tracks circus histogram; (E) SNP density; (F) Enhanced volcano plot; (G) KM survival curve; (H) Circle correlation pearson; (I) Correlation plot.

SRplot is developed for wet-lab biologists and designed to appeal to a wide range of use. This tool supports a wide variety of graphs commonly used in biomedical and bioinformatical publications, which are integrated into a user-friendly graphic user interface. As a new and comprehensive online tool, SRplot contains an extensive collection of more than 120 functions that cover genomics, transcriptomics, epigenomics, epi-transcriptomics, population genetics, evolutionary biology, differential gene expression, network data analyses and functions commonly used in COVID-19 publications (Fig 2). In detail, SRplot supports a number of graphing: 1) basic graph types, including bar plots, line plots, pie plots and scatter plots; 2) genome plots, including SNP density, Chromosome distribution and circus plots; 3) transcriptome plots, including heatmap, volcano plots, violin plots, bubble plots and chord plots; 4) epigenome plots, including metagene plots and motif plots; 5) clinical plots, including forest plots, KM plots and ROC curve. Our web tool allows users to simultaneously visualize data and prepare publishable graphs (Fig 3).

### Advantages

1, SRplot is freely available to non-commercial users.

2, This tool has a user-friendly graphical interface.

3, It is developed as an integrative toolkit. A wide variety of plots can be generated in SRplot.

4, It is an online tool that can be run using all modern Web browsers, including Internet Explorer, Google Chrome, Mozilla Firefox and Safari. The most computationally intensive plotting operations are performed at the server-side, so there are no strong requirements on the computing power of users’ machines. With our powerful server machine, most of the graphs can be created within seconds.

5, It is easy to perform the common modification operations, including shape, font, color, stroke width, and text.

6, The resulting graphs can be easily downloaded in common formats including PNG, TIFF, SVG and PDF.

7, Some commonly used databases, such as genomes/transcriptomes/GO/KEGG databases from human/mouse/rat and the TCGA data [8], are embedded on the website.

8, The website is updated promptly and continuously. Functions in SRplot have been improved, optimized and updated rely on feedback and recommendation from users, which includes over 46 000 stable users worldwide, many of whom are actively involved in the improvement of SRplot (Table 1).

**Table 1:** Comparison among platforms and software for data visualization and graphing.

Platforms/software	SRplot	Excel	Origin/Sigmaplot/Graphpad	TBtools	ggplot2/ matplotlib
Free of Charge	Yes	No	No	Yes	Yes
Types of graphs	****	***	****	**	*****
Out-of-the-box	Yes	No	No	No	No
Contains biomedical databases	Yes	No	No	No	No
Frequency of updating	Real-time	Regularly	Regularly	Regularly	Regularly
Learning curve	1	1	2	2	5
Changeable parameters	**	***	***	***	*****

### Input

We have provided the input data format for each plot to help the user to specify input parameters. Such input data files could be readily constructed in a document. Then the user should copy and paste the contents of the file into the SRplot input box. Standard formatting options, such as setting the plot name, figure size, font size and series colors, are all provided by the tool. Default settings for each item are offered. A number of options are provided for adjusting the parameters and final appearance of the graph. With a single click of the Submit button, the input data and specified style formats are combined to produce the resulting plot in real-time. If any further changes to the data and/or formatting styles are made, results are re-calculated and graphs are regenerated quickly. Meanwhile, data protection and privacy are guaranteed by offering secured data processing.

### Output

Tasks are performed on high-performance server and the resulting plots will be shown soon. If changing some of the settings, the plots can be quickly recreated. When a satisfactory version is produced, the plots can be easily downloaded by the user in a high-resolution bitmap (PNG or TIFF) or vector (PDF or SVG) format. All the graphs can be directly used in scientific publications.

## Discussion

Data visualization and graphing are important parts of biomedical publications. Some tools for this aim are coded in programming languages and based on command-line environments, which is difficult for an average user [4]. While some other software is commercial products, which always need hundreds of US dollars per license [1]. On the other hand, most of these tools or packages are designed for specific tasks, and several different tools are always needed to produce all figures for one single publication. To address these challenges, we integrated many commonly used functions into a single, freely accessible web tool, called SRplot. There are many significant advantages of our website, we helped address some of the learnability and usability issues related to many data visualization packages and software, and it saved a lot of time for our users.

Some of the data visualization and graphing work are heavy tasks and always have high computational requirements. When run locally, this kind of work needs long time to be finished. By using our powerful web server, most of the graphs can be generated within seconds. Moreover, plots can be easily created and modified using an intuitive user interface with SRplot. In contrast to regular graph generators, which usually produce uneditable figures, SRplot generates customizable features and interactive graphs full of editable elements. All graphs prepared in SRplot can be easily downloaded in both high-resolution bitmap and vector formats with publication quality to provide the user with maximum flexibility. In this regard, we believe SRplot will make data visualization and presentation much easier, much faster, and much more appealing to a much wider community of scientists, educators, and students.

A series of stellar functions has been used and validated by tens of thousands of users, making SRplot a handy and useful toolkit for biomedical researchers. The graphs prepared with SRplot have been featured in more than 550 peer-reviewed publications (Google Scholar data), including papers published in Nature Biotechnology [9], Nature Communications [10, 11], EMBO Molecular Medicine [12] and Diabetes [13]. Previously, we released the beta version (http://www.bioinformatics.com.cn), now we are going to present the updated version for the users worldwide (http://www.bioinformatics.com.cn/SRplot).

## Limitation

First, currently SRplot does not allow high dimensional data to be visualized on the website. This is mainly due to the performance issues: big data take long time to upload and run and, as a result, make the website run slowly and difficult to use. Second, to make better and more beautiful graphs, users may need to ask help from other graph editors, such as Inkscape and Adobe Illustrator. Third, SRplot is not an open-source platform at this moment, but it is freely accessible to all non-commercial users.

## Future developments

Certainly, the current version of SRplot is not the final web-based data visualization and graphing tool. In the future, our website will be updated promptly and continuously, and we expect to add more functionality regarding upstream and downstream data analysis and processing according to feedback and suggestions from users and progression in the biomedical field. Users can consult the SRplot website and the user manual for further information.
